# The emergence of porcine epidemic diarrhoea in Croatia: molecular characterization and serology

**DOI:** 10.1186/s12917-019-2002-x

**Published:** 2019-07-18

**Authors:** Dragan Brnić, Ivana Šimić, Ivana Lojkić, Nina Krešić, Andreja Jungić, Davor Balić, Marica Lolić, Dražen Knežević, Brigita Hengl

**Affiliations:** 1 Virology Department, Croatian Veterinary Institute, Savska Cesta 143, 10000 Zagreb, Croatia; 2 Veterinary Department Vinkovci, Croatian Veterinary Institute, Josipa Kozarca 24, 32100 Vinkovci, Croatia; 3 Croatian Agency for Agriculture and Food, Ivana Gundulića 36b, 31000 Osijek, Croatia

**Keywords:** Croatia, Domestic pig, ELISA, Outbreak, Porcine epidemic diarrhoea virus, S-INDEL

## Abstract

**Background:**

Porcine epidemic diarrhoea (PED) is an emergent/re-emergent viral pig disease (caused by the virus belonging to the *Coronaviridae* family, in specific the *Alphacoronavirus* genus) of global importance. Clinical presentation is characterized with acute diarrhoea, vomiting and dehydration in pigs of all ages, with a possible high mortality in suckling piglets. The disease emerged in the USA in 2013 causing heavy losses, and re-emerged in Europe in 2014, but with milder consequences.

**Results:**

In the spring 2016, PED-like symptoms were reported to be seen on an agricultural holding in Eastern Croatia; laboratory workup confirmed the Croatia’s first PED outbreak ever. Porcine epidemic diarrhoea virus (PEDV) strain responsible for the outbreak was of the S-INDEL genotype, much the same as other European PEDV strains. In 2017, a post-outbreak serology was carried out in three counties in Eastern Croatia, revealing seropositivity in pigs bred on four large industrial holdings (9.09%). The seroprevalence across PEDV-positive holdings was up to 82.8%. The latter holdings were unanimously managed by an enterprise that had never reported PED before.

**Conclusions:**

PED has emerged in Croatian pig population causing potentially considerable losses. The circulating strain was of the S-INDEL genotype. Serological workup proved PEDV spread to another four agricultural holdings, demonstrating the importance of not only external, but also internal biosecurity measures.

## Background

Porcine epidemic diarrhoea (PED) is a viral pig disease having a significant impact on pig production worldwide. Clinical presentation is characterized with acute diarrhoea, vomiting and dehydration in pigs of all ages, with a possible high mortality in suckling piglets [1]. The causative agent is porcine epidemic diarrhoea virus (PEDV), a positive single-stranded RNA virus belonging to the *Coronaviridae* family (genus *Alphacoronavirus*) [2]. The PEDV genome (~ 28 kb-long) comprises at least seven open reading frames (ORF1a, ORF1b, and ORF2–6) that encode four structural proteins (spike „S“, envelope „E“, membrane „M “and nucleocapsid „N“), two non-structural polyproteins (pp1a and pp1ab) and an accessory ORF3 protein [3]. The full-length S gene is known to be a suitable sequencing locus when it comes to the investigation into genetic relatedness and PEDV molecular epidemiology [4].

Even though PED was first recognized in the 1970s [5], it gained a lot of media attention in 2013, when a highly pathogenic PEDV variant caused a mortality of up to 100% in suckling US piglets, causing heavy losses in the first post-outbreak year [6, 7]. Two PEDV variants were proven to co-circulate in the USA at the time, i.e. S-INDEL (lower pathogenicity due to INsertions and DELetions seen in the S gene) and S2aa-del S gene variant (two amino acid deletions) [8, 9]. Moreover, recent reports confirmed the presence of PEDV strains with large deletions in S gene even in mixed infections caused by other S gene-containing PEDV variants [10, 11]. Since 2014, PEDV has re-emerged in Europe, but with milder consequences [12]. The PEDV strains behind the European PED outbreaks were of the S-INDEL genotype [12]. The only exception was the PEDV outbreak in Ukraine [13] caused by a highly virulent non-S-INDEL genotype strain, which, together with S-INDEL genotype strains and other PEDV variants, represents the group of newly-emerging PEDV strains that have appeared after 2010 [14]. On top of the above, there exists a group of classical PEDV strains, in circulation ever since the 1970s [14].

PEDV is a highly contagious virus easily spread by people, via transportation means, feed, aerosol and wild animals taking a faecal-oral route [4, 12, 15–17]. Therefore, strict biosecurity measures together with proper cleansing and adequate disinfection must be applied in order to block the entrance of PEDV into pig farms [4].

Croatia has been considered PED-free until the spring of 2016, when the suspicion on PED outbreak was first reported to the Croatian Veterinary Institute. This manuscript brings data on the very first PEDV outbreak in Croatia, together with the results of molecular characterization of the causative PEDV strain and post-outbreak serological workup.

## Results

### Detection of PEDV using molecular techniques

The presence of PEDV genome was confirmed in samples of piglets’ intestinal content using the real-time RT-PCR S gene (Sample 1 Ct = 13.67; Sample 2 Ct = 24.33) and N gene (Sample 1 Ct = 14.19; Sample 2 Ct = 31.36) protocol. Transmissible gastroenteritis virus (TGEV) and rotavirus A (RVA) were excluded as culprit agents since both of them tested negative.

### A sequence analysis revealed the presence of PEDV of the S-INDEL genotype

The NGS sequencing resulted in 42,168 merged reads (total reads *N* = 48,256), among which only four were mapped against the PEDV reference genome (KU297956) (data not shown). Furthermore, 347 reads were assigned to *Enterobacteriaceae,* while 30,869 showed significant sequence resemblance to the *Caudovirales*. Among *Caudovirales,* 20,194 reads were assigned to *Myoviridae* (*Shigella* phage Sp16, *N* = 14,913), while 10,377 were assigned to *Podoviridae* (*Escherichia* phage 172–1, *N* = 7718; *Escherichia* phage KBNP1711, *N* = 747; *Escherichia* phage ECBP2, *N* = 654; *Salmonella* phage, *N* = 83; and *Chronobacter* phage, *N* = 23).

Conventional three-step RT-PCR and the subsequent Sanger sequencing resulted in an almost complete S gene sequence (4137/4152 nt, 99.6%) and partial ORF3 gene sequence (36/675 nt, 5.3%) of the PEDV genome (4173 nt fragments in total). Phylogenetic analysis revealed the presence of PEDV of the S-INDEL genotype (Fig. 2), very similar to LT898414 and LT898444 strains witnessed in Germany (99.7/99.4% on nt/aa level), LT898433 and LT900502 strains witnessed in Austria (99.6/99.4–99.5% on nt/aa level), KU297956 strain witnessed in Slovenia (99.5/99.2% on nt/aa level), LT898436 strain witnessed in Romania (99.4/99.0% on nt/aa level), and KY111278 strain witnessed in Italy (99.4/99.1% on nt/aa level). The similarities with PEDV S-INDEL strains circulating in the USA were somewhat lesser (99.1–99.3% on nt level and 98.9–99.2% on aa level).

The analysis of additionally sequenced RdRp (565 nt) and M (524 nt) genome segments further confirmed that the virus responsible for the outbreak was PEDV (Fig. 3), not a TGEV-PEDV recombinant strain (swine enteric coronavirus, SeCoV).

### The Croatian PEDV strain was not adapted to grow in vitro

We attempted to grow the first Croatian PEDV strain in vitro, however the results were negative. The real-time S gene RT-PCR was positive for the inoculum (Ct = 27.25) and confirmed the first passage carryover (Ct = 35.53), but in the second passage the virus growth could not be detected (no Ct).

### Serological workup unmasked a larger proportion of the PEDV spread than first assessed

PEDV IgG ELISA test making use of a commercial IDVet ELISA kit resulted in 62 positive pigs (15.62%) and four positive agricultural holdings (9.09%) (Table 1).

**Table 1:** The results of serological workup

County	Blood samples	Holdings (large/backyard farms)	The number of PEDV Ab ELISA positive samples (%)	The number of PEDV Ab ELISA positive holdings (%)
Osijek-Baranja	115	23 (5/18)	1 (0.87)	1 (4.35)
Vukovar-Srijem	199	18 (5/13)	61 (30.65)	3 (16.67)
Brod-Posavina	83	3 (3/0)	0	0
Total	397	44 (13/31)	62 (15.62)	4 (9.09)

**Fig. 1:**
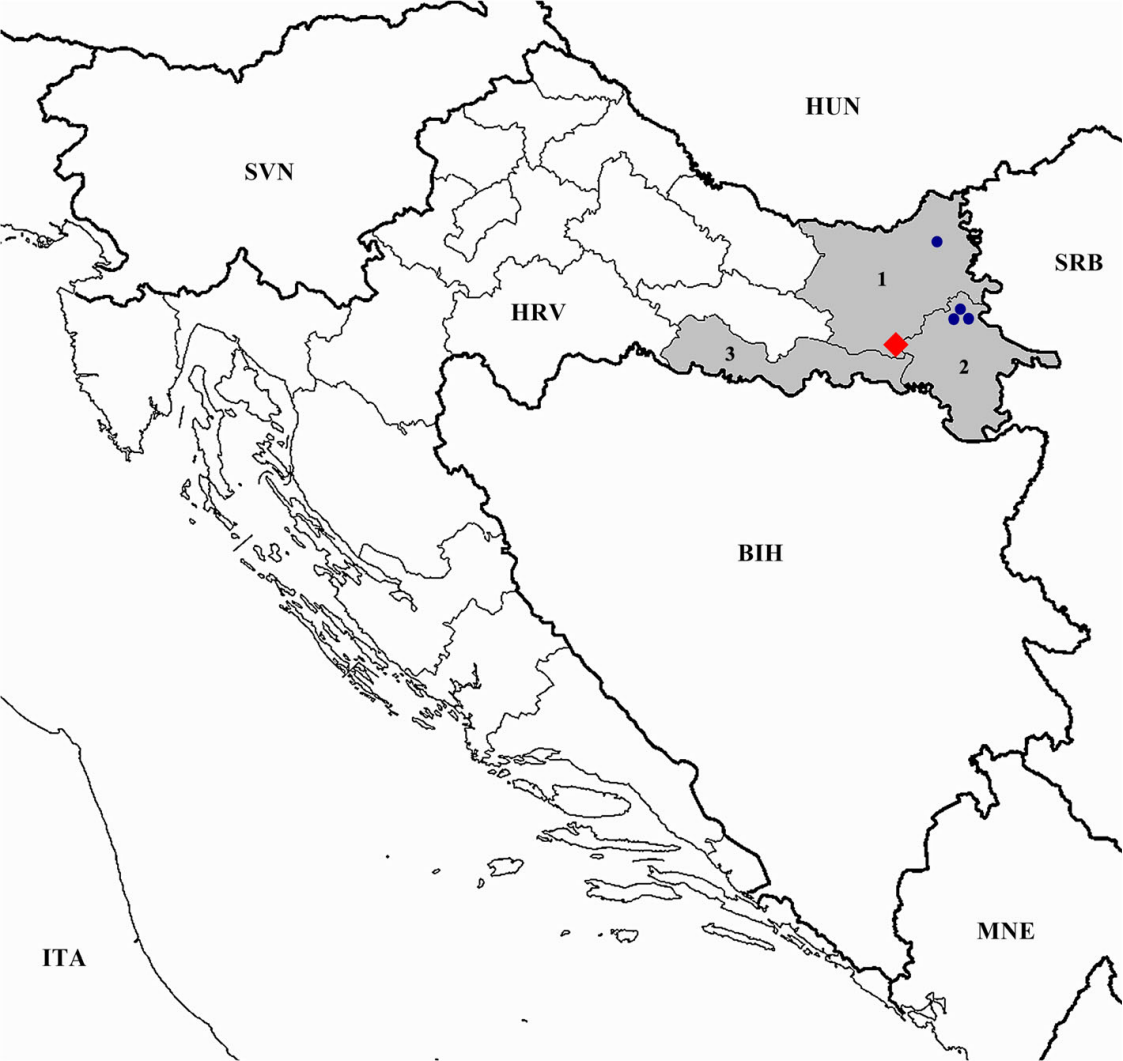
Geographical location of PEDV-positive holdings. The map shows the counties included into serological workup (light grey); 1 Osijek-Baranja County; 2 Vukovar-Srijem County; 3 Brod-Posavina County. The agricultural holdings hosting PEDV antibodies-positive animals are marked with dark blue dots, while the holding hosting the primary PEDV outbreak is tagged with a red rhombus. The map source is available at: https://commons.wikimedia.org/wiki/File:Croatia_location_map.svg (NordNordWest; CC BY-SA 3.0; https://creativecommons.org/licenses/by-sa/3.0)

Almost all PEDV IgG-positive pigs (*N* = 61) were finishers bred on three large neighbouring agricultural holdings seated in the Vukovar-Srijem County. Only one aborted sow originating from a large holding located in the Osijek-Baranja County tested positive (Fig. 1). The seroprevalence on three affected finisher farms was 55.2, 72.4, and 82.8%, respectively (the seroprevalence for farrow-to-wean farm with one positive sow was not calculated since only three samples were collected). These four affected holdings were managed by the same enterprise, which had never reported a suspicion on PEDV before. The sera of animals bred on the 2016 PEDV outbreak starting point were all negative, as were the animal sera retrieved on backyard holdings, which represented the majority of holdings tested within this frame.

## Discussion

**Fig. 2:**
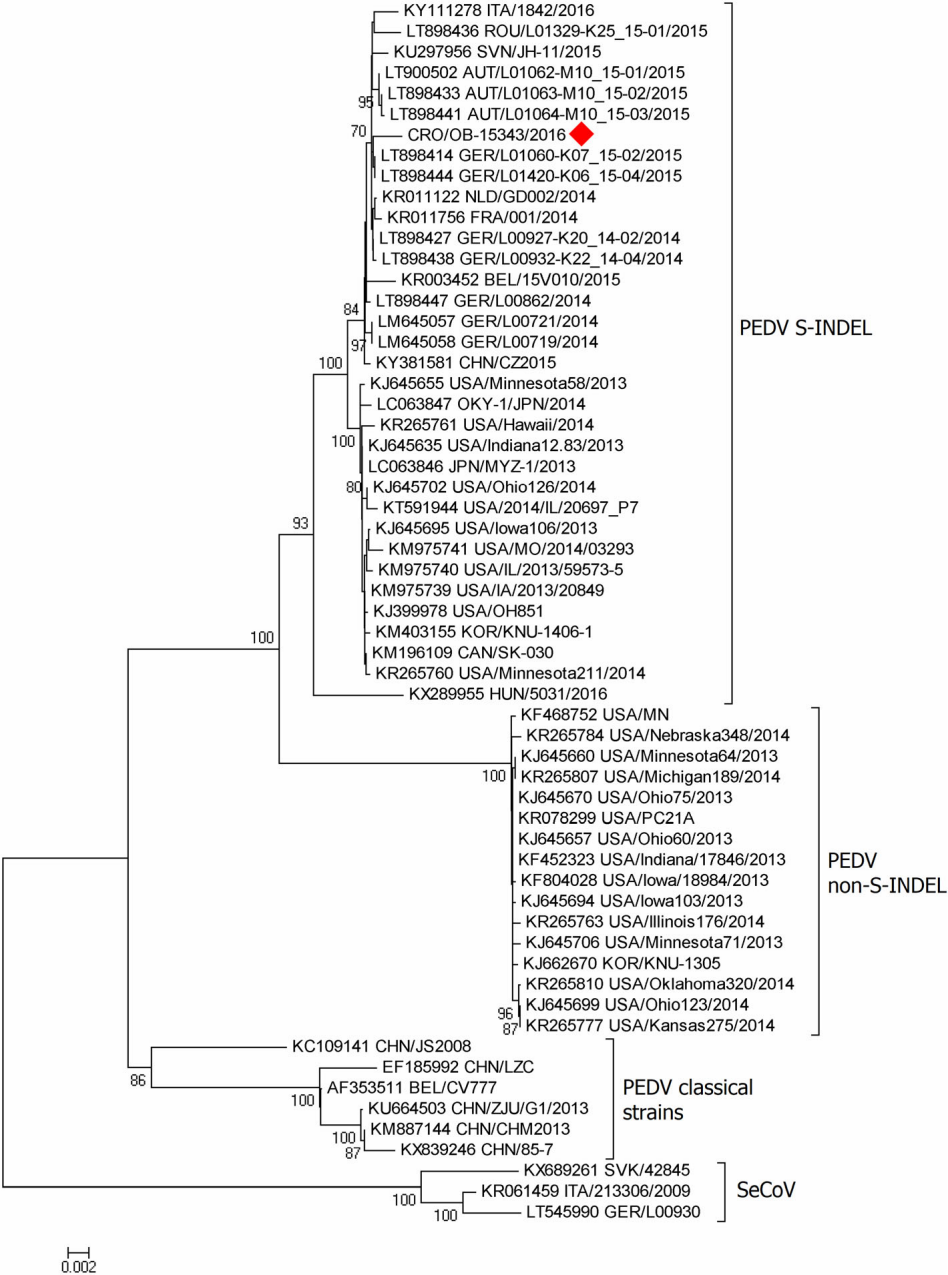
Phylogenetic relationship between the Croatian PEDV strain detected in 2016 and the selected reference strains (S gene segment-based). The phylogenetic tree was constructed from an almost complete PEDV S gene and a partial ORF 3 gene (4,173 nt in total) using a neighbour-joining method and the MEGA7 software with p-distances and 1000 bootstrap replicates (indicated adjacent to the nodes when > 70%). The first Croatian PEDV strain (CRO/OB-15343/2016) is tagged with a red rhombus. The GenBank accession numbers for the selected PEDV reference strains are designated within taxa. The scale bar represents the number of nucleotide substitutions per site

**Fig. 3:**
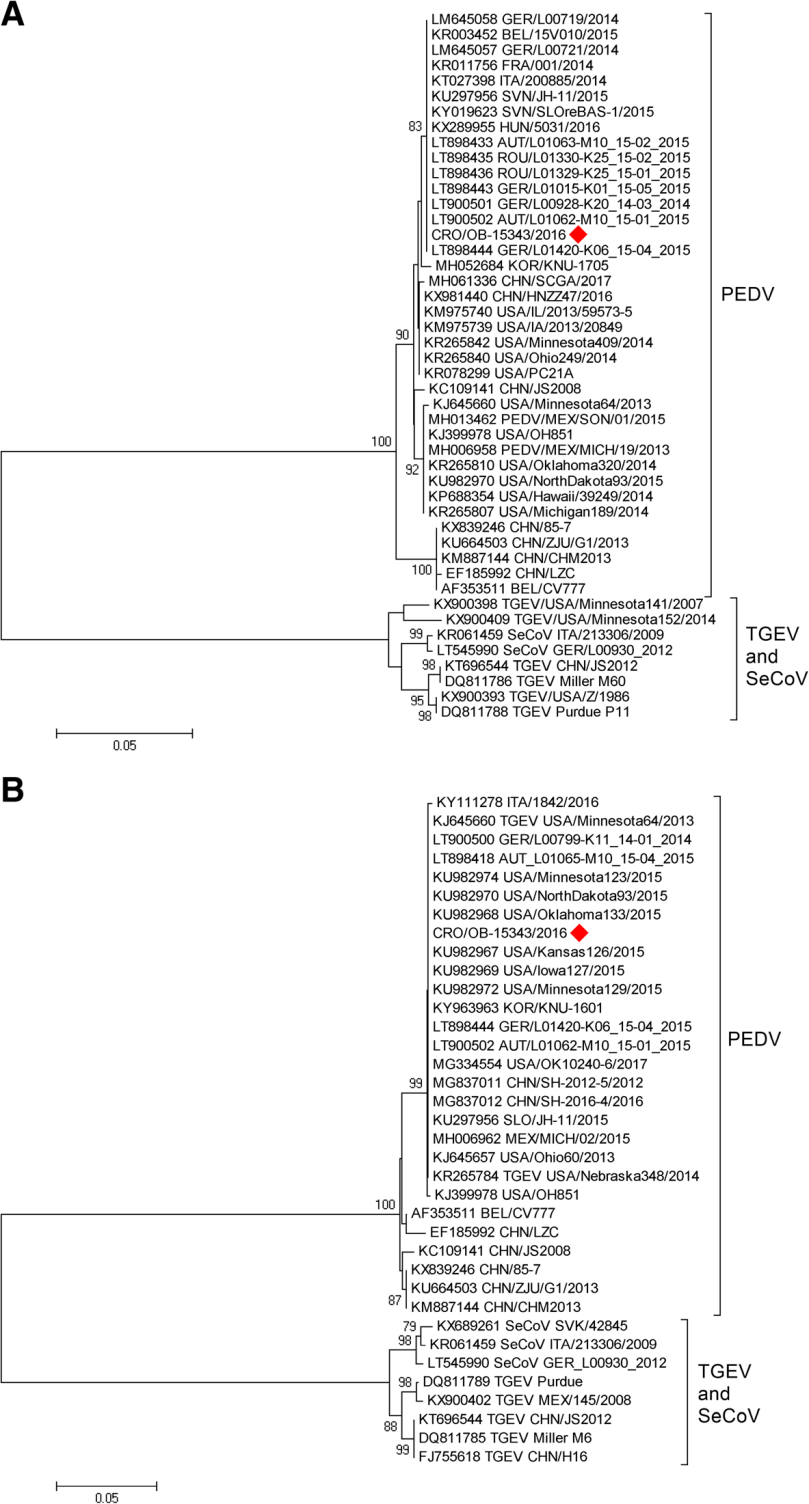
Phylogenetic relationship between the Croatian PEDV strain detected in 2016 and the selected reference strains (RdRp and M gene segments-based). The phylogenetic tree was constructed from partial RdRp gene (**a**) and partial M gene (**b**) segments using a neighbour-joining method and the MEGA7 software with p-distances and 1000 bootstrap replicates (indicated adjacent to the nodes when > 70%). The first Croatian PEDV strain (CRO/OB-15343/2016) is tagged with a red rhombus. The GenBank accession numbers for the selected PEDV reference strains are designated within taxa. The scale bar represents the number of nucleotide substitutions per site

Porcine epidemic diarrhoea has gained a lot of attention worldwide ever since 2013 due to the emergence of novel genotype strains in the USA. Croatia was considered PEDV-free until 2016, when the first outbreak was reported. This paper brings data on the first PED outbreak, molecular characterization of the detected PEDV strain and the subsequent serological workup. The first Croatian PEDV outbreak was reported later than in other western European countries [12], in the timeframe similar to Hungary [18] and Serbia [19]. However, the possibility of previous PEDV circulation cannot be excluded due to the general underreporting of gastrointestinal pig diseases in Croatia. The results of phylogenetic analysis showed the first Croatian PEDV strain to strongly resemble to the western European PEDV S-INDEL strains (Fig. 2), especially those detected in Germany and Austria (99.4–99.5% similarity on aa level) in 2015 [20, 21], which may suggest a trans-boundary route of spread. The amino acid sequence similarity with PEDV strains found in other surrounding countries was slightly lower; 99.0–99.2% with Romanian [20], Italian [22] and Slovenian [23] strains, and only 98.4% with the recombinant Hungarian [18] PEDV strain (sequencing data on the Serbian strain were not available). However, when it comes to the S gene-based comparisons, a possibly lower resolution should be taken into account as a limitation factor, which is not the case should the comparison be whole genome sequence-based. In the latter case, German, Austrian and Romanian PEDV strains referred to above, form a separate cluster different from 2014 German PEDV strains (strains found in Slovenia and Italy were not included) [20]. Unfortunately, our attempts to obtain the complete PEDV genome using the NGS, as well as those to isolate the virus in cell culture, were not successful, probably due to the condition of the sample which was subjected to several freeze-thaw cycles prior to sequencing. Even though the role of bacteriophages in the pathogenesis of gastrointestinal pig diseases has been hypothesized [24], we are of the opinion that their abundance suggested by NGS results does not compromise the role of PEDV as the main causative agent of this outbreak. We came to such a conclusion primarily due to the clinical presentation of the affected animals, epidemiological situation in the affected region, and the laboratory findings. Of note, the NGS was focused on virome; hence the involvement of bacteria and parasites in the clinical outcome might be underestimated. It is extremely difficult to determine the exact route of PEDV entry into a country based solely on sequencing and not detailed epidemiological data on human, animal and vehicle circulation. It is important to emphasize that Croatian pig production is characterized by a high, an ever increasing import volume of live pigs and pork, coming mostly from the EU member states, above all the Netherlands and Germany [25]. Therefore, there exists a possibility of co-circulation of other emerging enterotropic coronaviruses across Croatian pig population, primarily other PEDV S gene variants, porcine deltacoronavirus and TGEV-PEDV recombinant strains (SeCoV). The latter are known to circulate in Europe [26–28], but were excluded as the causative agent of the outbreak reported here (Figs. 2 and 3).

The second part of this study was oriented towards an indirect screening of PEDV circulation in pigs bred in three counties surrounding the location of the initial 2016 outbreak (Fig. 1). Therefore, in 2017 almost 400 sera of animals bred on 44 different holdings were tested, out of which 62 tested PEDV IgG- positive. The latter pigs were bred on four different holdings seated in two different Croatian counties (Table 1, Fig. 1). Data on PEDV Ab-seroprevalence in Europe are limited; the results are mostly negative or show low seroprevalence rates [12, 29], except for the Northern Italy where the seroprevalence is high [30]. All positive pigs-hosting holdings embraced by our study are managed by the same enterprise, which had never reported the disease before. However, in the late January 2017 the PEDV outbreak did occur, and was subsequently reported by the managing company [31]. Moreover, the losses were substantial, since three finisher and seven farrow-to-wean farms (out of nine) were affected (16,500 sows in total) with the 55 to 75%-mortality in suckling piglets [31]. Unfortunately, we couldn’t perform the molecular analysis on the PEDV strain responsible for the outbreak, since the company engaged a private German laboratory and the samples weren’t available to us (personal communication). In the present study, sampling timing was crucial for indirect detection of PEDV circulation on the affected farms (three finisher farms and one farrow-to-wean farm), since the antibodies against PEDV are known to be short-living [32]. The short life span of these antibodies might also be the reason behind negative results of the ELISA assay performed in pigs bred on the holding that hosted the primary 2016 outbreak, since the serology was done over a year later. Moreover, herd replacement practice and the possible introduction of naïve (PEDV Ab- negative) pigs that became a part of our random sample must also be taken into account. Even though the majority of holdings were of a backyard type, hence exercising poor biosecurity practices, they all (indirectly) tested PEDV-negative (negative IgG ELISA assay). As oppose to large, intense farming holdings, backyard holdings are characterized with lesser people/animal/vehicle circulation, known to be the major biosecurity risk for PEDV entrance and spread [30, 33]. Nevertheless, negative results must be interpreted with caution due to the abovementioned reasons coupled with well-known lower sensitivity of commercially available ELISA kits [34]. Despite the cross-reactivity between PEDV and TGEV that can take place when commercial Ab ELISA tests are used [35, 36], epidemiological evidence of a PEDV outbreak elaborated above [31] strongly suggest that the specificity of the Ab ELISA test used within the study frame was not an issue. Future studies on PEDV serology should include different diagnostic methods, so as to minimize the impact of test sensitivity/specificity variations. Moreover, detailed and representative epidemiological data obtained from the owners and veterinary officials are compulsory for the reliable interpretation of results.

## Conclusions

PEDV has emerged in Croatian pig population causing considerable losses. The circulating strain responsible for the first outbreak was of the S-INDEL genotype. Serological workup revealed PEDV presence on additional four holdings managed by the same enterprise that had never reported PED before, demonstrating the importance of not only external, but also internal biosecurity measures.

## Methods

### Outbreak description and sampling

In April 2016, the Croatian Veterinary Institute received two piglet carcasses (8–10 days old) for necropsy (voluntarily submitted by the owner). The agricultural holding of origin (a farrow-to-wean farm; 300 sows) was located in the Osijek-Baranja County (tagged by a red rhombus in Fig. 1). The owner reported a high prevalence of yellowish diarrhoea, vomiting and anorexia in suckling piglets, weaners and sows, and 20–30% mortality in suckling piglets. The necropsy showed severe dehydration and thin-walled intestines filled with yellowish watery to foamy fluid. Intestinal content (filling the small intestine) was taken for further molecular diagnostics.

### RNA isolation and real-time RT-PCR

A 10%-suspension of the intestinal content (*N* = 2) was prepared in the 199 Medium (Sigma Aldrich, Germany) and the supernatant was used for RNA isolation on an iPrep instrument using an iPrep PureLink Virus kit (Invitrogen, USA). The samples were tested for the presence of PEDV S gene [37], rotavirus A (RVA) VP2 gene [38] and transmissible gastroenteritis virus (TGEV) N gene [39] segments using a real-time RT-PCR. The primer/probe concentrations and cycling protocols were as recommended by the manufacturer of the QuantiFast Pathogen RT-PCR + IC kit (Qiagen, Germany) and the run was performed on a RotorGene-Q (Qiagen, Germany). The RVA protocol had one pre-step in terms of RNA denaturation by virtue of incubation for 5 min at 95 °C. The samples were also tested for the presence of PEDV N gene using a Sybr Green real-time RT-PCR (unpublished primers, kindly provided by Dr. Akbar Dastjerdi, APHA, UK) according to the instructions enclosed with the QuantiTect Sybr Green RT-PCR kit (Qiagen, Germany). The testing also made use of a RotorGene-Q.

### NGS and Sanger sequencing

Next Generation Sequencing (NGS) was performed on a MiSeq instrument (Illumina, USA) as described previously [40]. Sequencing data were analysed using the Geneious R10 Software (Biomatters Ltd., Auckland, New Zealand). Raw Illumina reads were trimmed for quality (phred quality score < 30) and short reads (< 75 bp) were discarded. The remaining paired reads were merged and non-merged reads/duplicates were discarded. Trimmed and merged reads were compared to the NCBI GenBank non-redundant nucleotide (BLASTn) with an E-value cut-off of 10^− 4^; the search was filtered so as to be restricted to the sequences in the database that correspond to the subset Viruses (taxid:10239). The BLAST output was used to create a taxonomic classification of the reads and contigs with the Megan 6.15.2. [41]. With the exception of the *Caudovirales* family, the obtained virus reads were extracted and further mapped against the GenBank viral non-redundant protein dataset (BLASTx) for confirmation. Furthermore, reads were mapped against the PEDV reference genome downloaded from the NCBI (KU297956).

Conventional amplification of the PEDV S gene was carried out in three steps. The first two steps included the implementation of PEDV-S1F/PEDV-S1R and PEDV-S2F/PEDV-S2R primer sets [42] and a Qiagen One-Step RT-PCR kit (Qiagen, Germany) under the thermal cycling conditions described by Chen et al. in 2014. In the third step of the PEDV S gene conventional amplification, the gap was closed by designing an additional primer set. The primers PEDV-S1/2F (5′-AACCATGTACAGCTAATTGC-3′) and PEDV-S1/2R (5′-ACCCATTGATAGTAGTGTCAG-3′) were employed with the abovementioned RT-PCR reagents under the above cycling conditions in the manner much the same as with the amplification that makes use of PEDV-S1F/PEDV-S1R primers, the only difference being a shorter elongation time (1 min). RT-PCR products were purified using a QIAquick gel or PCR purification kit (Qiagen, Germany) and sent to the Macrogen Europe (Amsterdam, the Netherlands) for direct Sanger sequencing in both directions.

In order to rule the circulation of TGEV-PEDV recombinant strains (swine enteric coronavirus, SeCoV) out, two additional conventional RT-PCR reactions for the amplification of RdRp and M gene segments were performed. To that effect, previously published primer sets that are pan-reactive to the representatives of the *Orthocoronavirinae* subfamily (RdRp gene) and to some members of the *Alphacoronavirus* genus (PEDV, TGEV, porcine respiratory coronavirus; M gene) were applied [26]. The primers were used with the abovementioned reagents under thermal cycling conditions as described by the Italian group [26].

### Phylogenetic analysis

Multiple sequence alignment and phylogenetic analysis were conducted using the MEGA7 Software [43] and neighbour-joining method with p-distance and 1000 bootstrap replicates. Nucleotide (nt) and amino acid (aa) pairwise identity matrix was calculated on previously aligned sequences using the BioEdit Version 7.0.5.3. [44]. The near-complete PEDV S gene sequence and the partial ORF3 gene sequence of the Croatian strain CRO/OB-15343/2016 was deposited in the GenBank under the accession number MK410092, while the fragments of PEDV RdRp and M genes were allocated the accession numbers MN046397 and MN046398, respectively.

### Cell culture

Virus propagation in vitro was attempted on Vero cells (ATCC® CCL-81™) in T_25_ flasks using the cell culturing protocol that does not imply inoculum removal (0.2 μm filtered supernatant of 10% -intestinal content suspension), as described by US scientists [45]. Virus growth was monitored using a real-time RT-PCR suitable for the detection of PEDV S gene, as described above. The material used for RNA isolation was a cell culture supernatant obtained after a single freeze/thaw cycle.

### Serological workup

Blood sampling of healthy pigs (i.e. pigs showing no signs of gastrointestinal disease or general signs of any given infectious disease) for the sake of serological workup was organized in the first half of 2017, together with sampling performed within the frame of the regular annual monitoring for classical swine fever and Aujeszky disease (carried out by the Ministry of Agriculture, the Veterinary and Food Safety Directorate). An agricultural holding that provided samples of aborted sows was included, as well. In total, 397 pig blood samples were retrieved from 44 randomly selected agricultural holdings (13 large and 31 small backyard holdings) seated in three eastern Croatian counties (Osijek-Baranja County, Vukovar-Srijem County, Brod-Posavina County) (Fig. 1, Table 1). The samples were taken from 204 sows and 193 finishers, the average number of samples retrieved on large holdings being 19.5 (range 3–32), and that harvested on backyard holdings being 3.7 (range 1–12). Among the holdings seated in the Osijek-Baranja County included into the study, the holding that hosted the primary 2016 PEDV outbreak was embraced, as well. In the majority of holdings (40/44), the sample size was tailored so as to be able to detect a 10%-seroprevalence within a 95% confidence interval, while on three holdings the sample size was set out so as to enable the detection of 20%-seroprevalence within a 95% confidence interval (one large farrow-to-wean holding with three samples of aborted sows didn’t meet these criteria). Blood samples were taken from the jugular vein into sterile tubes without anticoagulants, transported and stored in a cold environment (+ 4 °C). The sera were separated from cellular elements by virtue of coagulated blood centrifugation for 15 min at 1,000 g (blood clots thereby being rimmed with a sterile glass stick so as to facilitate separation) and stored at − 20 °C prior to testing. All sera were tested for the presence of specific IgG antibodies against PEDV using an ID Screen® PEDV Indirect ELISA test (IDVet, France).

## Data Availability

The datasets used and/or analyzed within the frame of the study can be provided by the corresponding author upon a justified request.

## Abbreviations

Ab: Antibody
BLAST: Basic Local Alignment Search Tool
BLASTn: Basic Local Alignment Search Tool for searching nucleotide databases using a nucleotide query
BLASTx: Basic Local Alignment Search Tool for searching protein databases using a translated nucleotide query
ELISA: Enzyme-Linked Immunosorbent Assay
NCBI: National Center for Biotechnology Information
NGS: Next Generation Sequencing
PED: Porcine Epidemic Diarrhoea
PEDV: Porcine Epidemic Diarrhoea Virus
RT-PCR: Reverse Transcriptase Polymerase Chain Reaction
RVA: Rotavirus A
SeCoV: Swine Enteric Coronavirus
S-INDEL: PEDV strain with insertions and deletions seen in the S gene
TGEV: Transmissible Gastroenteritis Virus
